# In search of time to bring the message on social media: Effects of temporal targeting and weather on digital consumers

**DOI:** 10.3389/fpsyg.2022.1042714

**Published:** 2022-11-16

**Authors:** Gunwoo Yoon, Cong Li, John Juyoung Choi

**Affiliations:** ^1^Department of Marketing and Entrepreneurship, University of Northern Iowa, Cedar Falls, IA, United States; ^2^Department of Strategic Communication, University of Miami, Coral Gables, FL, United States; ^3^Spicy Tribe Inc, Los Angeles, CA, United States

**Keywords:** contextual marketing, social media, field experiment, temporal targeting, weather

## Abstract

Marketers always incline to deliver advertising messages to the right consumer at the right time. Yet, the question of when exactly should such a persuasive message be sent to a consumer remains elusive in the existing literature. The current study aims to address this research question within the theoretical framework of contextual marketing. The authors argue that contextual information such as time and weather can be used to design more effective mobile advertising campaigns on social media. The results of a field experiment in cooperation with a local restaurant suggest that ads delivered at consumers’ pre-decision stage (i.e., non-meal time) are more effective than those delivered at the decision stage (i.e., meal time) to increase consumer spending on the dining-in service. Furthermore, unpleasant weather conditions (i.e., less sunlight) are found to improve the effectiveness of advertising on consumer spending on mobile app food delivery orders. Overall, the authors open future research avenues by demonstrating how and why the two contextual factors, time and weather, influence digital consumer behavior.

## Introduction

The emergence of social media and mobile technologies has transformed marketing and changed consumer consumption (Andrews et al., 2016). Particularly, the estimated number of mobile device owners worldwide is over five billion (McCarthy, 2019). People touch their mobile phones thousands of times per day and these devices almost function like “an extra limb” on the human body (Naftulin, 2016). Many time-consuming tasks now can be accomplished by a few simple actions such as typing, tapping, and swiping the phone’s screen. For example, instead of going to a restaurant, people can order food delivery *via* a mobile app, and online food delivery is a rapidly growing segment with the global revenue topping $107 billion in the year of 2019 (Statista, 2000).

Given that people are constantly connected online with their mobile devices, companies should consider this a valuable opportunity to stay in touch with their consumers “24 h a day, 7 days a week, anywhere on the planet” (Kenny and Marshall, 2000, p. 123). Yet, how to use rich mobile data (e.g., temporal and spatial information) to design and deliver more effective marketing communications to consumers is a critical research question to be further studied (Luo et al., 2014). Mobile technologies are ubiquitous in nature and personable, making them suited for targeted and context-driven promotional messages. Suppose mobile data enable advertisers to know who their consumers are, and where they are at a particular moment, will the advertising message, then, be ready for the right consumer at the right time in the right place? Unfortunately, the existing advertising literature has not offered clear answers to this question. The question of when exactly is the “right” time to deliver an ad to a consumer, for example, remains ambiguous. To shed light on this research direction, we conduct a field experiment. We have partnered with a newly launched restaurant (located in Buena Park, California), developed product promotion and brand messages on Facebook as mobile advertising, and collected daily transaction data from restaurant diners and delivery app orders. Here we primarily examine how the time of day and weather jointly influence the advertising effect on the restaurant business performance (i.e., revenue). Before presenting the experimental results, we first explain the conceptual framework.

## Conceptual overview

### Personalization and contextual marketing theory

Previous studies have discussed how to personalize a message for an individual, such as using his/her demographic characteristics, personality traits, or preferences to construct the message (Teeny et al., 2021). Although these approaches differ in how specifically a personalized message will be created, the fundamental idea is the same: building a connection between the message and the message receiver’s identity (Li et al., 2020). Because a personalized message speaks to one or more aspects of the message receiver’s identity, it tends to generate a feeling of relevance, which consequently yields favorable effects in attitude and behavior (Kalyanaraman and Sundar, 2006).

It is worth noting that a feeling of relevance can not only be triggered by the recognition of the message recipient’s identity in the message content, but also by the communication context (Wang et al., 2014). For example, Li (2019) demonstrated that a message might be perceived as either relevant or irrelevant for a person, depending on the schema that is primed for that person before the message evaluation. Following this logic, if a message “matches” a particular feature of the communication environment where the message is delivered to the receiver, it should be perceived as relevant, and thus generate favorable persuasion effects. Also, Ketelaar et al. (2017) conducted an experiment by engaging participants in a virtual supermarket grocery shopping task. It was found that when the stimulus ad was congruent with the shopping environment (i.e., the advertised product was presented on the shelf), participants’ tendency to choose the advertised product was significantly higher.

Such an idea of personalizing messages based on the communication environment or setting looks particularly attractive in a mobile world, as new mobile technologies enable advertisers to access consumers’ spatial and geographical information (Luo et al., 2014). Kenny and Marshall (2000) once proposed a theory called “contextual marketing,” which suggests that marketers should use the power and reach of the Internet to present the most relevant information to consumers when they need it. Similarly, Lian et al. (2019) labeled such targeting strategies that incorporate consumers’ contextual information (e.g., time, location, and behavior) as “contextual targeting.” The central idea of this context-related strategy is to utilize the contextual information to figure out consumers’ “point of need” because they will be most receptive to advertising information at that point (Kenny and Marshall, 2000). To apply this reasoning to the current study, at what point (e.g., time of day) will consumers need advertising messages from a local restaurant?

### Construal level theory and temporal targeting

People can see and experience only the here and now; thus, anything that is not directly experienced at the present moment is distal (Liberman et al., 2007). According to such a psychological distance concept or the construal level theory (Trope and Liberman, 2003), temporal distance influences people’s responses to future events by systematically changing the way they construe those events. In particular, people tend to form more abstract representations of distant-future events (i.e., high-level construal) but more concrete representations of near-future events (i.e., low-level construal). Due to these distinctions, information with different levels of orientation to the future is expected to generate different persuasion effects.

Trope and Liberman (2003) argued that a high-level construal process focuses on desirability considerations of actions (e.g., “why” an action should be performed), while a low-level construal process is more about the feasibility of actions (i.e., “how” an action can be performed). They further showed that feasibility concerns (e.g., time constraints) are more prominent in planning the near future than the distant future. For instance, in one of their studies, participants were asked to identify how many hours they would spend on both academic and non-academic activities during either “next week” (i.e., the near future) or “a week a year from now” (i.e., the distant future). It was found that the total number of hours planned was lower for the near future than that for the distant future; that is because participants took the time constraints (e.g., multiple activities may occur at the same time, thus they need to be planned at the expense of each other) into account more for the near future.

This effect of time constraints (i.e., as a form of temporal/psychological distance) is especially relevant to the food business when consumers choose to dine in because they need to plan a physical trip to the restaurant. As elaborated by Lian et al. (2019), physically going to a restaurant to dine in involves a “travel cost,” which includes not only monetary cost but also opportunity cost of time. Therefore, whether a restaurant ad can influence consumer decision on visiting the restaurant depends on the tradeoff between the travel cost and the attractiveness of the ad. For example, Fong et al. (2015) showed that consumers may be willing to shop at a geographically non-proximal location against a proximal one, if the promotion offers a deeper discount. However, to order food delivery *via* mobile apps does not engage physical travel, thus the effect of time constraints should be less prominent for an app order.

To examine the effects of contextual and temporal marketing on consumers, we focus on two specific time frames in this study: meal time and non-meal time. In particular, meal time refers to traditional time windows when people have meals, whereas non-meal time is outside those windows. For restaurant consumers, these two frames of time can be considered the pre-decision stage (i.e., searching for information, evaluating alternatives) and the decision stage (e.g., making a purchase decision), respectively. An ad delivered at the pre-decision stage should have a stronger persuasion effect than that delivered at the decision stage for dining-in services because it allows more time for travel planning (Lian et al., 2019). In contrast, since ordering food delivery by mobile apps does not have the same level of time constraints as with dining-in, ads delivered at either the pre-decision stage or the decision stage are unlikely to produce significantly different results. Based on this reasoning, the following hypothesis is proposed:

*H1:* Ads delivered at a non-meal time will generate more consumer spending on dining-in, but not on app orders, than ads delivered at a meal time.

### Effect of weather

As described earlier, a key distinction between dining-in and ordering food delivery *via* mobile apps is the “travel cost.” In theory, travel is constrained not only by time, but also by weather. Although the influence of weather on human behavior has been well documented in several domains such as finance, it is less discussed in the marketing literature. How weather influences the way people process advertising information, for example, largely remains unknown. We aim to fill this theoretical gap by examining how weather conditions influence the effectiveness of advertising on consumer decisions on dining-in at a restaurant or ordering food delivery *via* mobile apps.

According to Murray et al. (2010), weather can affect consumer behavior in three general ways. First, bad weather (e.g., rain, snow) tends to keep people at home. Second, people are likely to consume certain products at a higher or lower rate under particular weather conditions (e.g., more purchases of ice cream in summer than in winter). Third, weather can influence a person’s psychological state (e.g., mood), which subsequently affects his/her consumption behavior. For example, Agarwal et al. (2020) documented a “sunshine effect,” estimating that a one-unit increase in local sunshine (i.e., sky cover as a proxy for sunshine) would increase the daily spending of an average consumer by around $0.41. Shafi and Mohammadi (2020) found that change in sky cloud cover from zero to full would likely reduce investors’ risky investments in equity crowdfunding by 10%–15%. Li et al. (2017) compared the effects of sunny, rainy, and cloudy weather conditions on consumers’ purchase responses to promotions. It was revealed that sunny weather generated about 1.21 times more responses than cloudy weather, while rainy weather led to about 0.9 times fewer responses. Govind et al. (2020) showed that pleasant weather conditions (e.g., more sunlight, less cloudiness, and lower precipitation) tend to generate positive emotions, which will consequently reduce hedonic consumption (e.g., purchasing chocolates and cookies), and this effect is more prominent with women than men.

Given that pleasant weather conditions elicit a positive emotional state and also reduce the level of constraints on travel, we expect advertising messages delivered to consumers in such conditions to drive more dining-in behaviors. On the contrary, unpleasant weather conditions keep consumers at home, thus more food delivery orders *via* mobile apps are anticipated. Based on this logic, we propose the following hypothesis:

*H2:* (a) Ads delivered under pleasant weather conditions (e.g., more sunlight) will generate more consumer spending on dining-in, while (b) ads delivered under unpleasant weather conditions (e.g., less sunlight) will generate more consumer spending on app orders.

## Materials and methods

As previous research showed that a restaurant setting is suitable for examining the effect of context marketing (Lian et al., 2019), we partnered with a newly launched restaurant for data collection in this study. The restaurant is mid-sized in scale and located in Buena Park (Orange county), California. We carried out the field experiment in collaboration with a digital marketing agency, Spicy Tribe, and collected 50-day transaction data from restaurant diners (9,650 transactions) and delivery app orders (800 transactions) who were unaware of the current study. With the digital marketing agency, we also developed product promotion and brand messages on Facebook on behalf of the restaurant owner. Because food advertising typically involves visual appeals, we believe advertising through social media to consumers is more appropriate for the current study than other forms of mobile advertising such as short message services. To implement temporal targeting, 50 different messages were posted on Facebook during the 95-day experimental period. Particularly, half of randomly selected promotional messages were posted on Facebook at a meal time (i.e., lunch, 11 AM–1 PM; dinner, 5 PM–7 PM) and the other half were posted at a non-meal time (i.e., anytime, except for the meal time). We should note that the restaurant solely used promotional posts on Facebook for its marketing activities during the experimental period; no other advertising or promotional activities were performed.

### Model specification and data

To estimate the effect of temporal targeting and weather, the following linear regression model was formulated: *Y* = *α*_0_ + *α*_1_*Ttargeting* + *α*_2_*Ccontext* + κ + γ + τ + ε, where *Y* is the outcome variable (e.g., sales from dine-in consumers in natural log), Ttargeting is a dummy indicating temporal targeting (coded as 1 if a promotional message was posted on Facebook at the meal time and 0 otherwise), Ccontext is a set of weather-related variables (e.g., precipitation), and α is a coefficient vector. The model also consists of several time-invariant variables and controls; κ, γ, and τ represent social media metrics (e.g., daily post impression), message features (e.g., type of message), other environment fixed effects (e.g., covid cases, advertising expenditure in natural log), respectively, and ε is an error term. After specifying the model, we started collecting data. The research period was 95 days, and we ran the field experiment from 3rd January to 6th April 2021. Fifty randomly selected days were used for temporal targeting interventions: 25 days for temporally proximal targeting (i.e., meal time) and 25 days for temporally non-proximal targeting (i.e., non-meal time).

We also collected daily data on consumers’ dining and delivery transactions. We particularly recorded Facebook post-related features like linguistic concreteness and metrics. Afterward, we used LIWC 2015 (Linguistic Inquiry and Word Count) software to measure message concreteness. Previous linguistics literature (Snefjella and Kuperman, 2015; Pan et al., 2018) has suggested that concrete (abstract) language tends to use verbs (vs. adjectives), numbers (vs. non-specific qualifiers), and past-focused words (vs. future-focused). We treated language concreteness as an aggregate score and calculated it for each product promotion or brand message on Facebook. To be specific, we followed a well-established normalizing procedure (see Pan et al., 2018 for details). That is, we utilized six different word categories from LIWC (i.e., verbs, adjectives, numbers, non-specific quantifiers, past-focused, and future-focused), normalized each word category score, summated normalized scores of concreteness and subtracted the normalized scores of abstractness. As a result, we developed the measure for linguistic concreteness of the ad message on Facebook, with a mean of 0.0013 and a standard deviation of 2.43.

We also collected social media metrics derived from our temporal targeting intervention on Facebook such as the number of times a post entered a person’s screen a day and the number of people who engaged with a page daily. Further, we collected the cost of each social media post and attempted to control for exogenous variation in advertising effects. We also supplemented those data with contextual weather data from the National Centers for Environmental Information^1^ and publicly accessible data on COVID-19.^2^ Table 1 shows all variables, their definitions, and summary statistics.

**Table 1:** Descriptive statistics.

Variables	Definition	M	SD	Min	Max
Sales_dine	Sales from dine-in consumers	3701.86	1245.54	1740.75	6615.52
Sales_deli	Sales from delivery app orders	421.19	146.19	168.91	903.5
Adexp	Monetary cost for each Facebook post	62.64	29	56	200
Dimpression	Daily post impression, the number of times a post entered a person’s screen a day	5998.9	2874.88	958	13,282
Dusers	Daily engaged users, the number of people who engage with a post on a page	158.7	102.46	35	631
Mconcrete	Message concreteness, a continuous concreteness-abstractness measure with six normalized lexical category scores from LIWC	0.001	2.43	−7.74	6.16
Pre	Average precipitation level (inches)	0.04	0.11	0	0.49
Wsp	Average wind speed (MPH)	5.34	2.65	1.9	13.8
Cloud	Average sky cover	2.3	2.42	0	8
Ntests	Number of COVID-19 tests reported (Orange county, CA)	14727.84	7326.53	3,298	32,288
Cases	Daily COVID-19 positive cases received (Orange county, CA)	737.86	910.52	62	4,080
Deaths	Daily deaths reported (Orange county, CA)	22.36	18.95	0	66
Ndoses	Number of doses administered (Orange county, CA)	18794.2	8482.76	3,509	39,430
	Type			Obs	%
Ttargeting	Temporal targeting (dummy)		Meal time	25	50
			Non-meal time	25	50
Mtype	Message type (dummy)		Photo	27	54
			Video	23	46
Sunshine	The amount of sunshine (dummy)		High	32	64
			Low	18	36
Covidtier	Guidelines for opening businesses to mitigate spread of COVID-19 (dummy)		Widespread	45	90
			Substantial	5	10

## Results

### Initial evidence and regression analyses

To ensure that any observed effects were not due to Facebook post-driven appeals or evaluation dimensions, we first conducted a pretest to confirm all Facebook posts we used did not significantly differ in their persuasiveness and general likeability. Forty-four undergraduate students participated in this pretest (M_age_ = 21.16, SD_age_ = 0.91, 21 females); they were asked to review and evaluate 10 randomly selected Facebook posts (out of 50) with three 7-point scale items ranging from 1 = not at all to 7 = very much (to what extent do you like the post/do you think the post is expressive/do you think the post is assertive, Cronbach’s alpha > 0.78). A series of independent t-tests showed that the Facebook posts did not differ in terms of participants’ responses, *ts* < 0.89, *ps* > 0.38.

To test the hypotheses, OLS regression examined the relationship between our temporal targeting intervention and sales outcomes.^3^ Our main outcome measures were sales from dine-in consumers and delivery app orders. The results in Table 2 show a negative and significant relationship between temporal targeting and sales from dine-in consumers (*α*_1_ = −0.364, *p* < 0.01). This relationship persists even when controlling for a number of alternative explanations, including social media metrics, message features, covid-related, and advertising expenditure variables. However, this effect is not observed when predicting the sales from delivery app orders (*α*_1_ = −0.578, *p* = 0.13). These results support the hypothesized relationship predicted in H1, indicating that temporal proximity (i.e., close vs. far depending on the length of time from when promotional messages get delivered/posted to the point of consumption choice) indeed influences mobile advertising effectiveness but only for those who require time for their purchases. Furthermore, in H2a we predicted an effect of pleasant weather on dine-in consumers; yet, this hypothesis was not supported (all *p*s > 0.23). However, we found a negative and significant relationship supporting H2b; the sunshine was negatively related to the sales from delivery app orders (*α*_2_ = −0.27, *p* < 0.05).

**Table 2:** Regression analysis results.

	DV: Sale from dine-in consumers (in natural log)		DV: Sales from deliver app orders (in natural log)	
	b	SE	b	SE
**Experimental intervention**				
Ttargeting	−0.364^**^	0.105	−0.578	−0.151
**Social media metrics**				
Duser	0.0003	0.0006	−0.001	0.0007
Dimpression	−0.00001	0.00003	0.00005	0.00003
**Message**				
Mtype	−0.07	0.104	−0.281	0.141
Mconcrete	0.008	0.025	−0.044	0.06
**Climatic contexts**				
Pre	−0.045	0.443	−0.945	0.856
Wsp	−0.024	0.019	−0.062	0.015
Cloud	0.009	0.034	−0.061	0.079
Sunshine	0.079	0.171	−0.27^*^	0.427
**Control variables**				
Covid-related	Included			
Adexp	−0.049	0.212	−0.48	0.382
Number of transactions	9,650		800	
R-squared	0.39		0.37	

* *p* < 0.05;

** *p* < 0.01.

### Robustness checks

We took a few additional steps to test the robustness of our results. First, we performed tests to check the presence of heteroskedasticity. Our examination of the residuals and the tests from the regression models suggested that there was no heteroskedasticity problem (Breusch–Pagan/Cook–Weisberg test, *p* > 0.44; White’s test, *p* > 0.43). Thus, we did not use any parameters with robust standard errors. Second, the variance inflation factor (VIF) was also tested to see whether our models had any multicollinearity issues for selection of explanatory variables. The mean VIF from the regression models was 3.12 (ranged from 1.33 to 7.67), which was below the acceptable threshold of 10. We concluded that the two regression models were linear combinations of independent measures.

Next, to further check the robustness of our regression results, we adopted regression machine learning and ran more advanced data analysis techniques with python to apply to the same data. Regression machine learning is different from traditional regression as it first trains a proposed model by using a training dataset and a test dataset and then predicts estimates with the unseen/unobserved test dataset (Aziz et al., 2022). It also automatically removes exploratory variables if they lack power. We utilized the linear regression (LR), decision tree regressor (DTR), random forest regressor (RFR), gradient boosting regressor (GBR), support vector regressor (SVR), and multi-layer perceptron regressor (MLPR) as implemented in Scikit-learn.^4^ We should note that our data were randomly divided into training and testing sets as 80% and 20%, respectively, and each machine learning regression is performed independently. We obtained consistent results from regression machine learning; no coefficients differed in sign or significance. Yet, we argue that these advanced machine learning techniques provided testing prediction accuracy. Particularly, the root mean square error (RMSE) and the mean absolute percentage error (MAPE) showed that the prediction testing accuracy levels with the proposed regression models were higher than 87.97% (LR > 94.69%; DTR > 95.27%; RFR > 96.1%; GBR > 96.17%; SVR > 96.4%; MLPR >87.97%). Altogether, our models and data from the field experiment present robust estimation results and demonstrate the effect of temporal targeting and weather on two types of consumer spending, dining-in and mobile ordering.

## Discussion

The effect and efficacy of digital marketing have been extensively explored in various fields, and previous literature has shown that it can, directly and indirectly, influence sales (Stephen, 2016; Kannan and Hongshuang, 2017; Yoon et al., 2018). In accordance with that perspective, researchers proposed the contextual marketing theory more than 2 decades ago and recommended marketers to shift their mobile marketing focus from content to context. More recent empirical research has investigated the effects of contextual factors (e.g., location, time, or product characteristics, Bart et al., 2014; Luo et al., 2014); yet, academic research that examines how consumers’ real-time context affects mobile marketing effectiveness is surprisingly scarce (Fong et al., 2015). More importantly, in real-world marketing practice, there is no consistent guiding theory such that some advertisers choose to deliver messages evenly across hours while others select specific hour blocks for advertising (Phang et al., 2019).

Given that the effectiveness of marketing communications is context-dependent, reaching out to consumers (with the right message) at the right time is critical. We address the key research question of what exactly is the right time to deliver an ad to a consumer by carrying out a randomized field experiment. Particularly, we attempt to offer some insights into the very intriguing question of timing by testing how different times of a day, in addition to the weather of that day, influence the effect of advertising within a restaurant business setting. We argue that time and weather are two critical contextual variables that will affect how consumers assess advertising information, and this effect is especially salient in a mobile world. Because of the wide use of mobile phones and online ordering *via* mobile apps, we identify two types of consumer behavior in the restaurant business setting, one being on-site dining and the other being food delivery by mobile apps. We suggest that advertising campaigns on social media should be crafted for these two behavioral types in different ways because the “context” associated with each of these behaviors is uniquely different. For example, the dining-in behavior might be more constrained by time and weather than the app ordering behavior. The results of this study demonstrated that ads delivered at consumers’ pre-decision stage tend to be more effective for the on-site service as they give consumers sufficient time to conquer their travel constraints. In contrast, ads delivered under unpleasant weather conditions tend to enhance the travel constraints, thus function more effectively for the mobile app food delivery orders.

Our results help us to quantify the relationship between a restaurant’s contextual targeting and its sales from dine-in consumers as well as delivery orders. We show that ads delivered at consumers’ pre-decision stage (i.e., non-meal time) tend to be more effective for the on-site service as they give consumers sufficient time to conquer their travel constraints. However, this pattern is not observed from remote/delivery orders. When it comes to the effect of weather on contextual marketing, we find that ads delivered under unpleasant weather conditions lead to the travel constraints, and thus, function more effectively for the mobile app food delivery orders. To summarize, differential temporal targeting and weather effects are largely consistent with our theoretical predictions, confirming that a mobile advertising’s persuasion effect can be different if it is delivered to the same message recipient but at different times. The current research extends the context marketing literature by advancing a novel way to think about contextual marketing: temporal and climate targeting. The results also suggest that for mobile marketers and restaurant managers, temporal and climate targeting represent a new contextual setting for measuring the effectiveness of context-specific ad messages.

We should note that, as argued by Grewal et al. (2016), time, weather, and physical location can influence consumer responses to advertising because these contextual factors may activate different needs. The key to using contextual information for personalization is to create a feeling of relevance, which is highly situational (Wang et al., 2014). Different from rather stable demographic and personality characteristics, the contextual feature associated with a person tends to change from moment to moment, thus the “right” time of communication often suggests a “match” between the message and the message receiver’s context. Unfortunately, prior research on this message-context match is very limited (Teeny et al., 2021), and how to deliver the right message to the right person at the right time is still an unresolved mystery.

To our best knowledge, the current study is among the first to examine how time and weather, as two contextual factors, influence advertising effectiveness. We hope to highlight the importance of contextual information in developing and implementing advertising campaigns. Using the restaurant business as an example of examination, we reveal that contextual marketing is a dynamic process and the goal is to align the advertising message with a temporary context in which the message recipient is. We argue that a particular context will activate or de-activate a certain consumer need (e.g., a sunny day may weaken people’s need to use mobile apps to order food delivery). With the help of new mobile technologies, creating highly personalized messages to match people’s needs has become more actionable than ever (Teeny et al., 2021).

There are a few study limitations and future research directions that should be addressed. First, the current study offers some exploratory findings on how time and weather affect consumer responses to mobile advertising in a field experiment. Although these field data point to real consumer behaviors, we are unable to rule out alternative explanations for these behaviors besides the effect of time and weather in the strictest sense. Previous empirical studies have speculated some benefits of real-time targeting with locational, temporal, and promotional implementations. More future studies on the effect of temporal targeting and weather are thus called for, especially with other research methods.

Second, this study reveals how consumers react differently to an advertising message, depending on the specific stage of consumer decision (i.e., pre-decision vs. decision). However, how long each stage may endure may be subject to the decision importance. For instance, the pre-decision stage may last much longer for planning a birthday party luncheon than planning for an everyday work lunch. Also, for those different stages of consumer decision-making process, we were not able to warrant that the advertising message on Facebook was processed or clicked by all customers. Future research is needed to examine the effect of decision stage on contextual marketing with different decision types.

Third, though our models capture the effects of temporal targeting and climate contexts on consumer behavior, we solely rely on experimental and non-experimental variations in one restaurant’s sales data, and thus, the research does not have a large number of data points. Also, the field experiment here took place in just one selected city in California. We assume that weather might have different impacts on food consumption decision-making (i.e., for both dine-in and delivery app consumers) if we chose some cities located in Midwest or targeted people in the East Coast region. That is because sensitivity to weather variability in the U.S. varies across geo-locations and culture (Lazo et al., 2011). We want to acknowledge that there might be a risk of having an over-fitting problem; future research would benefit from collecting big data across different types of restaurants (or other businesses) in more diverse locations.

Finally, in discussing the weather effect, this study categorizes weather conditions into either pleasant or unpleasant. It is worth pointing out, however, that what is pleasure or unpleasant weather may be a subjective judgment. For example, more sunshine is generally considered good weather, but too much sunshine with high levels of temperature and humidity may be deemed unpleasant. Future research should explore these nuanced differences in testing the effect of weather on advertising.

New mobile technologies have enabled advertisers to use context-related information for designing more effective personalization strategies. However, how to incorporate contextual information such as time and weather into advertising practice to improve persuasion effects remains under-explored in the literature. Our results suggest that time is a significant predictor of advertising effect on on-site dining, while weather appears to be a critical factor to influence advertising effect on food delivery orders by mobile apps. The current study offers some preliminary findings from the field experiment to direct future research.

## Data availability statement

The raw data supporting the conclusions of this article will be made available upon request, without undue reservation.

## Author contributions

GY developed the idea and outline for the study with input from CL, GY, CL, and JC collected and analyzed the data. GY, CL, and JC commented on the revisions of this study, and GY and CL review and edited the manuscript. All authors listed substantially contributed to the study, and they all read and approved the final version of this study.

## Conflict of interest

JC was employed by company Spicy Tribe Inc. The remaining authors declare that the research was conducted in the absence of any commercial or financial relationships that could be construed as a potential conflict of interest.

## Publisher’s note

All claims expressed in this article are solely those of the authors and do not necessarily represent those of their affiliated organizations, or those of the publisher, the editors and the reviewers. Any product that may be evaluated in this article, or claim that may be made by its manufacturer, is not guaranteed or endorsed by the publisher.

## Footnotes

^1^ www.ncdc.noaa.gov

^2^ https://covid19.ca.gov

^3^An independent-samples *t*-test on overall sales showed that temporal targeting (regardless of the interventions: meal time and non-meal time) plays a significant role in determining overall sales (in US dollars); temporal targeting indeed increased overall purchase amounts (M_temporal targeting_ = 4123.05, SD_temporal targeting_ = 184.48 vs. M_control_ = 3536.44, SD_control_ = 145.33), *t* (93) = 2.46, *p* < 0.05, *d* = 0.51, 95% CI = [0.10, 0.91]. However, weather did not generally influence overall business performance, *t*(93) = 0.18, *p* = ns.

^4^ https://scikit-learn.org
